# BRCA1 mutations in ovarian cancer and borderline tumours in Norway: a nested case–control study

**DOI:** 10.1038/sj.bjc.6602199

**Published:** 2004-10-12

**Authors:** T Bjørge, A K Lie, E Hovig, R E Gislefoss, S Hansen, E Jellum, H Langseth, K Nustad, C G Tropé, A Dørum

**Affiliations:** 1 Department of Pathology, The Norwegian Radium Hospital, Oslo N-0310, Norway; 2 Department of Tumour Biology, The Norwegian Radium Hospital, Oslo N-0310, Norway; 3 Janus serum bank, Institute of Clinical Biochemistry/Department of Clinical Chemistry, Rikshospitalet University Hospital, Oslo, Norway; 4 The Cancer Registry of Norway, Montebello, Oslo N-0310 Norway; 5 Central Laboratory, The Norwegian Radium Hospital, Oslo N-0310, Norway; 6 Department of Gynaecologic Oncology, The Norwegian Radium Hospital, Oslo N-0310, Norway

**Keywords:** cancer genetics, epidemiology, ovarian cancer, borderline tumours, case–control study

## Abstract

The aims of the present study were to find the frequency of the most common BRCA1 mutations in women with ovarian tumours identified from a population-based cancer registry and in the general population, to estimate the relative risk of ovarian tumours among the mutation carriers, and to explore the value of using CA125 as a prediagnostic test. The study was designed as a nested case–control study within a cohort mainly consisting of participants in population-based health examinations. The data files of The Cancer Registry of Norway and the Janus serum bank were linked to identify cases with ovarian cancer and borderline tumours. Hereditary BRCA1 mutations were determined using archived serum samples and capillary electrophoresis. Altogether 478 ovarian cancer patients and 190 patients with borderline tumours were identified, and 1421 and 568 matching controls were selected. Odds ratios (OR) of developing ovarian cancer and borderline tumours in the presence of BRCA1 mutations and CA125 level were derived from conditional logistic regression models. Among the 478 ovarian cancer patients, 19 BRCA1 mutations were identified (1675delA, 1135insA, 816delGT and 3347delAG), none among the patients with borderline tumours. Only two of the 1989 controls were BRCA1 mutation carriers (0.10%). The risk of ovarian cancer among the mutation carriers was strongly elevated (OR=29, 95% CI=6.6–120). CA125 was a marker for ovarian cancer, but the sensitivity was low. This study showed that BRCA1 mutation carriers have a very high risk of ovarian cancer. However, since the prevalence of BRCA1 mutations in the Norwegian population was low, the proportion of ovarian cancers due to BRCA1 mutations seemed to be low, about 4%. The sensitivity of using CA125 only as a screening test for ovarian cancer was low.

Ovarian cancer is the sixth most common neoplasm among women worldwide, and is a disease of affluent societies (Parkin, 2001). The Nordic countries, except for Finland, exhibit some of the highest incidence rates in the world. In Norway, the incidence of ovarian cancer has increased moderately since 1970 (Bjørge *et al*, 1997). Ovarian cancer patients have a poor prognosis. In Norway, the prognosis of patients with ovarian cancer has improved steadily since the 1950s, the 5-year relative survival reaching about 40% in 1993–1997 (Cancer Registry of Norway, 2003).

Ovarian cancer has a hereditary proportion exceeding 10% in some populations (Risch *et al*, 2001; Narod and Boyd, 2002). The risk of ovarian cancer conferred by a BRCA1 mutation by the age of 70 years is reported to be between 36 and 66% (Thompson and Easton, 2002; Antoniou *et al*, 2003). Women with BRCA1 mutations are diagnosed at a younger age than noncarriers, and most tumours in the mutation carriers are of serous histology (Stratton *et al*, 1997; Risch *et al*, 2001). The frequency of BRCA1 mutations in the general population has been estimated at 0.06–0.14% (Ford *et al*, 1995; Whittemore *et al*, 1997).

In Norway, BRCA1 founder mutations are assumed to be caused by the rapid population expansion after the Bubonic plague (Black death) in the 14th century, which reduced the population by approximately 60% (Dørum *et al*, 1997). Previously, the two founder mutations (1675delA and 1135insA) have been shown to account for about half of all BRCA1 cases and for one-third of hereditary breast-ovarian cancers in Norway (Borg *et al*, 1999). By including 816delGT and 3347delAG, these four founder mutations have been calculated to account for 68% of the Norwegian BRCA1 mutation carriers (Møller *et al*, 2001a, 2001b). The penetrances of 1675delA and 1135insA with respect to breast and ovarian cancer have been reported to be high (Dørum *et al*, 1999a).

CA125 has demonstrated its value in monitoring the treatment of patients with ovarian cancer (Makar, 1993). This marker has also in combination with ultrasound been promising in screening for early detection of ovarian cancer (Jacobs *et al*, 1999). At present, there has been no randomised trial of screening using serial CA125 levels and ultrasound in BRCA1 mutation carriers, and observational cohort studies have been disappointing (Narod and Boyd, 2002).

In Norway, the Janus serum bank was established in the early 1970s, and a population-based cancer registry has been in operation since the early 1950s. In the present study, we used these data sources to find the frequency of the most common BRCA1 mutations in women with ovarian cancer and borderline tumours, and in the general Norwegian population. We also aimed at estimating the relative risk of ovarian cancer among the mutation carriers. Further, we aimed to explore the value of using CA125 as a prediagnostic test.

## MATERIALS AND METHODS

### The Janus project

The Janus serum bank was initiated in 1973, and contains approximately 700 000 serum samples, stored at −25°C, from about 330 000 donors (The Norwegian Cancer Society, 2004). The specimens have been collected from individuals who participated in county health examinations mostly for cardiovascular diseases, and from blood donors. The participants in the health examinations were recruited from several counties in various parts of Norway. The blood donors were from the Red Cross Blood Donor Centre in Oslo.

### The Cancer Registry of Norway

Since 1953, the Cancer Registry of Norway has received information on all cancer patients in the population. The reporting system is based on pathology and cytology reports, clinical records and death certificates, and provides information about site, histological type and stage of disease at the time of diagnosis. Also, the 11-digit individual identification number allocated to every resident of Norway is reported. The registration of ovarian cancers is regarded as practically complete and of high quality (Tingulstad *et al*, 2002). Through 1992, registration was based on a modified version of ICD-7. Since 1993, ICD-O has been the basis for coding.

### Identification of cases and controls

The data files of the serum bank and the cancer registry were linked to identify cases with ovarian cancer and borderline tumours that had donated serum at least 1 month before diagnosis. The ovarian cancers and the borderline tumours, with corresponding controls, were selected separately. If there were several serum samples available per case, the last (youngest) sample before diagnosis was chosen. Three women were selected from the cohort as controls for each case. The controls were individually matched for age at serum sampling (±3 years), storage time (±1 year), county of residence and blood donor status.

Initially, 534 ovarian cancers and 215 borderline tumours were identified. Serum samples were not available for five ovarian cancers and three borderline tumours. Hence, 1587 and 636 matched controls were identified for the ovarian cancers and borderline tumours, respectively. Serum samples were not available for 15 controls. Thereafter, 529 ovarian cancers and 212 borderline tumours with 1574 and 634 matching controls, respectively, were left.

Diagnostic biopsy specimens were received from 17 pathology laboratories for histopathologic review from 514 of the cancer cases and 201 of the borderline cases. More than 70% of the relevant histopathologic material already existed at the Norwegian Radium Hospital; the remaining was sent to us as double set of unstained slides, which were stained with haematoxylin and eosin (HE). All specimens were re-evaluated blindly by one of the authors (AKL). The tumours were classified and graded according to the criteria defined by the Atlas of Tumour Pathology/AFIP (Scully *et al*, 1998).

After re-evaluation of the histology diagnoses, 25 of the cancer cases were classified as metastases and three as benign tumours, and hence excluded from the cancer study. A total of 17 cases were classified as borderline tumours, and hence transferred to the borderline study. After re-evaluation of the borderline patients, 16 tumours were classified as benign, three as metastases and one as uncertain (whether metastasis or primary tumour). All these were excluded from the study. Of the remaining borderline cases, nine were classified as cancers and transferred to the cancer study. After the histological re-evaluation, 478 ovarian cancer cases and 190 borderline tumours with 1421 and 568 matching controls, respectively, were left for analysis. The cancer cases were diagnosed from 1975 to 2001 (median: 1994), and the borderline tumours were diagnosed from 1974 to 2000 (median 1995). The matching criterion on age at serum sampling was expanded for 23 controls (range: 3.5–3.9 years).

Only small differences in the median age and time between serum sampling and diagnosis were observed between cases and controls and between the two study populations (Table 1

**Table 1:** Characteristics of the study population

	**Ovarian cancer**		**Borderline tumour**	
	**Cases (*n*=478)**	**Controls (*n*=1421)**	**Cases (*n*=190)**	**Controls (*n*=568)**
*Age at diagnosis (years)*^a^				
Median (range)	52 (33–78)	51 (30–77)	49 (26–76)	49 (23–78)
				
*Time between serum sampling and diagnosis (years)*^a^				
Median (range)	7.2 (0.1–26)	7.2 (−0.5–26)	7.1 (0.1–23)	7.2 (−0.5–23)
				
*Histology*				
Serous	251 (53%)		96 (51%)	
Mucinous	28 (6%)		86 (45%)	
Endometrioid	76 (16%)		3 (2%)	
Clear cell	53 (11%)			
Mixed epithelial	24 (5%)			
Undifferentiated	20 (4%)			
Granulosa cell	15 (3%)			
Other	11 (2%)		5 (3%)	
				
*BRCA1 mutations*				
816delGT	1	0	0	0
1135insA	5	1	0	0
1675delA	8	1	0	0
3347delAG	5	0	0	0
Any mutation	19	2	0	0
				
*CA 125*				
Median (range)	14 (0–100)	10 (0–100)	15 (0–100)	11 (0–100)
No. positive (>35 U ml^−1^)	71	79	35	45

a For controls, the date of diagnosis for the belonging case was used.

).

### Laboratory methods

#### BRCA1

Mutation analysis for four founder mutations, 1675delA, 1135insA, 816delGT and 3347delAG, was carried out. Owing to limited amount of serum, some sera were not tested for all the founder mutations. One case and four controls were tested for one founder mutation only, 16 cases and 30 controls were tested for two, 91 cases and 301 controls were tested for three, while 560 cases and 1654 controls were tested for all four mutations. In this study, persons with at least one of the four founder mutations were termed BRCA1 mutation carriers, irrespective of the number of mutations tested.

A detailed description of all methodological aspects is published elsewhere (Ekstrøm *et al*, 2004). Briefly, 5 *μ*l of serum were aliquoted to 96-well plates (Axygen, Tamro Medlab AS, Oslo, Norway), followed by microwave boiling at 1200 W for 4 min. PCR master mix (40 *μ*l) was added to each well, mixed and subjected to thermal cycling with primer pair specific parameters. PCR products were analysed using an unmodified MegaBACE 1000 (AmershamBiosciences Uppsala, Sweden) capillary sequencing instrument for fragment lengths. Samples were injected electrokinetically from the PCR plates, with no post-PCR cleanup, and subjected to electrophoresis at 60°C with a field strength of 145 V cm^−1^ for 30 min.

#### CA125

The serum volume available was 50 *μ*l. This was diluted manually 10 times in assay buffer 0.05 mol l^−1^ Tris, 0.15 mol l^−1^ NaCl, 0.02 mmol l^−1^ DTPA, 0.005 g l^−1^ Tatrazine, 10 g l^−1^ Germall II, 0.001% Triton X-100, 5 g l^−1^ BSA, 0.5 g l^−1^ bovine IgG and 15 mg l^−1^ MAK33, pH 7.8. MAK33, a mouse IgG1 monoclonal from Roche, was heated to 60°C for 10 min (Bjerner *et al*, 2002). A modified version of our fully automated in-house CA125 assays was established as follows. In an Auto-DELFIA from Perkin-Elmer Wallac (Turku, Finland) microtitre plates coated with streptavidin (Perkin-Elmer Wallac) was incubated with 125 *μ*l antibody K93 (2 *μ*g ml^−1^ of biotinylated (Fab′)_2_ in assay buffer) for 20 min and the plate washed three times. Assay buffer (50 *μ*l) and diluted serum samples (50 *μ*l) were added, incubated for 90 min and the plate washed three times. Europium-labelled antibody K101 (100 ng in 100 *μ*l assay buffer) was added, incubated for 20 min and the plate washed six times before adding 200 *μ*l enhancement solution and reading delayed fluorescence. Samples were run as singlicates and standards and controls in duplicate. Two controls were run twice on each microtitre plate. The low control measured in 27 assays gave a mean of 33 kU l^−1^ with 5.3% CV (total error). The high control gave mean 159 with 5.8% CV. A cutoff level of 35 kU l^−1^ was used as in our in-house assay. The in-house assay has been thoroughly evaluated for interference from heterophilic antibodies (Bjerner *et al*, 2002). The antibodies have been studied in several workshops and the routine assay, which is essentially similar to the modified assay except for the use of 25 *μ*l undiluted serum as sample, behaves well as judged from international quality control programmes (Nustad *et al*, 1996, 2002).

All laboratory analyses were performed with coded samples.

### Statistical analyses

Odds ratios (OR) and their 95% confidence intervals (95% CI) were derived from conditional logistic regression models. Throughout the analysis, a significance level of 5% was used. The data were analysed using the program package EGRET (Statistics and Epidemiology Research Corporation (SERC Inc.), 1999). For CA125, receiver-operating characteristic (ROC) curves were calculated, using SPSS (SPSS Inc., 2001), to evaluate the cutoff used and the usefulness of the test. Curves were calculated for different age groups (<50 years and ⩾50 years) and for different time intervals before diagnosis (<0.5, 0.5–1.4, 1.5–2.4, 2.5–4.9, 5–9 and 10 years or more).

### Ethics

The Regional Ethics Committee and the National Institute of Data Inspection approved the study.

## RESULTS

### BRCA1 mutations

Among the 478 ovarian cancer patients, 19 BRCA1 mutations were identified (4.0%) (Table 1), and no mutation was discovered among the 190 patients with borderline tumours. The frequency of BRCA1 mutations in ovarian cancer patients younger than 50 years was 7.4% (13 of 176). Above 50 years, the frequency was 2.1% (six of 283). Two mutations (1135insA and 1675delA) were identified among all the controls (*n*=1989). One of these controls developed ovarian cancer 3 years after being used as a control.

The median age at diagnosis among the ovarian cancer mutation carriers was 48 years (range: 46–54 years), 52 years (range: 33–78) among the noncarriers. In all, 74% of the tumours among the ovarian cancer mutation carriers were of serous histology, 52% among the noncarriers. Seven of the ovarian cancer mutation carriers (37%) were from the county of Rogaland in the southwestern part of Norway (816delGT, 1675delA and 3347delAG).

There was an increased risk of developing ovarian cancer among the mutation carriers (OR=29, 95% CI=6.6–120) compared with the noncarriers. Both the two mutation carriers among the controls had two primary cancers. One had cancers of the uterine corpus and breast, diagnosed after serum sampling. The other mutation carrier among the controls was also included in the study as a case. In addition to the ovarian cancer diagnosis (at the age of 48 years), she had breast cancer. Among the other 1419 controls, 145 had at least one cancer diagnosis. Four of the 19 ovarian cancer mutation carriers had breast cancer as well, diagnosed prior to the ovarian cancer.

### CA125

Among the 478 ovarian cancer patients, 71 (15%) were CA125 positive at serum sampling. Of the controls, 79 (6%) were positive. A higher risk of ovarian cancer was observed in women with elevated CA125 (OR=3.1, 95% CI=2.2–4.4). Restricting the analyses to cases with serum sampling less than 2 years prior to diagnosis and matched controls gave a higher OR (OR=13, 95% CI=4.2–37) (Table 2

**Table 2:** ORs and 95% CIs of ovarian cancer and borderline tumour according to BRCA1 mutations and CA125

	**Ovarian cancer**			**Borderline tumour**		
	**Case/Ctrl**	**OR**	**95% CI**	**Case/Ctrl**	**OR**	**95% CI**
*BRCA1*^a^						
Negative	459/1419	1.0	Referent	190/568	1.0	Referent
Positive	19/2	29	6.6–120	0/0	—	—
						
*CA125*						
Negative	407/1342	1.0	Referent	155/523	1.0	Referent
Positive (overall)	71/79	3.1	2.2–4.4	35/45	2.5	1.6–4.1
Lag time (years)^b^						
0–1	56/167	13	4.2–37	23/69	32	4.0–260
2–3	68/201	2.1	0.8–5.5	31/92	1.9	0.7–5.6
4–5	75/224	1.5	0.6–4.2	30/90	6.0	1.5–24
6–7	77/231	2.3	0.9–5.5	27/81	1.7	0.6–4.7
8–9	72/216	1.7	0.6–4.4	20/60	0.4	0.0–3.4
10–14	99/292	3.3	1.5–7.2	47/140	1.6	0.5–4.9
15+	31/90	13	1.4–130	12/36	1.5	0.1–17

OR=odds ratio; CI=confidence interval.

a 816delGT, 1135insA, 1675delA and/or 3347delAG.

b For controls, time between serum sampling and diagnosis for the belonging case.

). Restricting the analysis to cases with serum sampling less than 5 years prior to diagnosis also increased the OR (OR=4.0, 95% CI=2.3–6.9).

Only two of the 19 ovarian cancer mutation carriers had elevated CA125 levels. However, only three of these 19 women had serum sampling less than 5 years prior to diagnosis.

A total of 35 of the patients with borderline tumours (18%) were CA125 positive at serum sampling, 45 of the controls (8%). A higher risk of borderline tumours was observed among women with elevated CA125 (OR=2.5, 95% CI=1.6–4.1). Restricting the analyses to cases with serum sampling less than 2 years prior to diagnosis and matched controls again gave a higher OR (OR=32, 95% CI=4.0–260).

A higher risk of ovarian cancer was observed in CA125-positive women older than 50 years (OR=3.3, 95% CI=1.5–7.1), not among the younger women (Table 3

**Table 3:** ORs and 95% CIs of ovarian cancer and borderline tumours according to CA125 by age groups

	**Ovarian cancer**		**Borderline tumour**	
	**OR**	**95% CI**	**OR**	**95% CI**
*All*				
CA125 positive				
Case less than 40 years old	1.1	0.3–3.5	0.8	0.2–3.0
Case 40–49 years old	3.5	2.3–5.4	2.8	1.6–4.8
Case 50+ years old	3.3	1.5–7.1	12	1.3–110
				
*CA125 measurements less than five years prior to diagnosis*				
CA125 positive				
Case less than 40 years old	—	—	4.4	0.4–51
Case 40–49 years old	4.3	2.1–8.8	6.6	2.7–16
Case 50+ years old	5.2	1.9–14	3.0	0.2–48

OR=odds ratio; CI=confidence interval.

). A higher risk of borderline tumours was observed among CA125-positive women older than 40 years, but most pronounced in women older than 50 years (OR=12, 95% CI=1.3–110).

**Figure 1:**
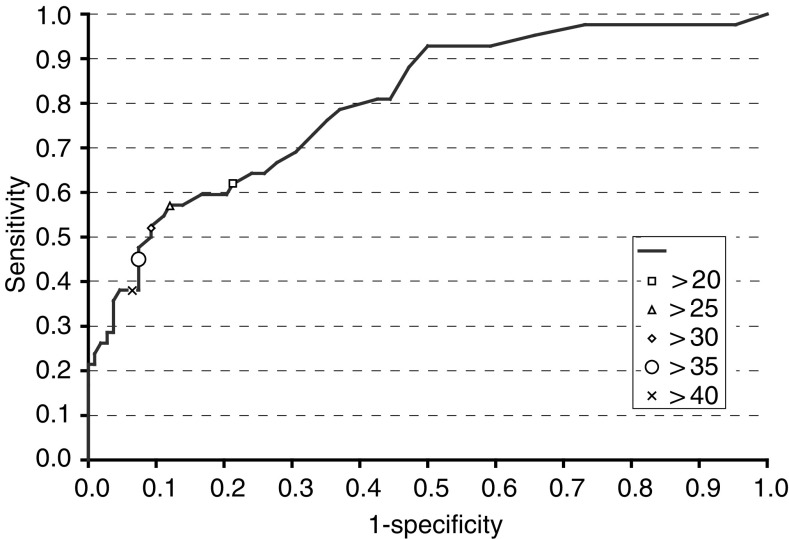
Receiver operating characteristic curve for CA125 as a prediagnostic test for ovarian cancer less than 1.5 years prior to diagnosis. The area under the curve (ROC area) is 0.80 (95% CI: 0.72–0.88). The points corresponding to different cut offs (kU l^−1^) are marked.

The estimated ROC curves revealed that CA125 was a fair or good test for ovarian cancer only with a time perspective of about 1.5 years. Therefore, we only present figures with this perspective. For women under the age of 50 years, the area under the ROC curve (ROC area) was 0.76 (95% CI: 0.65–0.87), indicating that CA125 is a fair test for this perspective. For women at or above the age of 50 years, the test was good with an ROC area of 0.87 (95% CI: 0.77–0.97). Figure 1

shows the ROC curve with time perspective of 1.5 years for all women combined. The sensitivity connected to the chosen cutoff was 0.45, while the specificity was 0.93.

## DISCUSSION

In the present seroepidemiological study, we showed that only 4% of the ovarian cancers in Norway were due to BRCA1 mutations (1675delA, 1135insA, 816delGT and 3347delAG), and none of the borderline tumours. Only two of the 1989 controls were BRCA1 mutation carriers (0.10%). The risk of ovarian cancer among the mutation carriers was, however, strongly elevated. CA125 was a marker for ovarian cancer, but the sensitivity was low.

We have previously reported that known hereditary mutations in the BRCA gene can efficiently be analysed in serum samples (Ekstrøm *et al*, 2004). With a primer design producing less than 100 bp products, we were able to achieve amplification success rates ranging from 83 to 98%, depending on primer pair. The success rates obtained with these primers are in fact not much lower than that observed from larger series with extracted full-length DNA samples (data not shown). By selecting such short product sizes, it thus appears clearly feasible to apply this technology on serum samples that have been stored frozen for many years.

Even though the registration of ovarian cancers in Norway is regarded as practically complete and of high quality (Tingulstad *et al*, 2002), we have shown that it is important to re-evaluate the histology in population-based studies like this. The intra- and interobserver reproducibility of the classification of ovarian tumours is relatively low (Stalsberg *et al*, 1988). The specimens were selected from the time period 1972–2000 and from 17 different laboratories. One pathologist reviewed all histology diagnoses to ensure important diagnostic groups. After revision of the diagnoses, we had to omit 7% of the cases since they did not meet the criteria for ovarian cancer or borderline tumours.

To date, relatively few studies on BRCA mutations are population based (Risch *et al*, 2001; Sarantaus *et al*, 2001). In most studies, women are selected on the basis of a family history positive for cancer, or for early age at onset. In the present population-based study, the cases were selected by linkage of the data files of a population-based cancer registry to the files of a serum bank where the donors mainly were participants in population-based health examinations.

In the present study, we were not able to adjust for parity or oral contraceptive use. However, a larger set of data is necessary to adjust for these variables in a meaningful way.

Large differences in the BRCA1 mutation frequencies among ovarian cancer patients have been reported. The highest frequencies have been shown among Ashkenazi Jews (35 and 27%; Boyd *et al*, 2000; Moslehi *et al*, 2000) compared to 4.7 and 3.5% in the Finnish (Sarantaus *et al*, 2001) and British population (Stratton *et al*, 1997), respectively. Previously, it has been reported that 3% of Norwegian ovarian cancers are caused by BRCA1 1675delA or 1135insA (Dørum *et al*, 1999b). We observed a low prevalence of BRCA1 mutations in this population-based case–control study. However, the penetrances of these mutations are among the highest reported (Dørum *et al*, 1999a), which were confirmed in this study. We only tested for the four most common founder mutations, previously being reported to account for 68% of the BRCA1 mutation carriers in Norway (Møller *et al*, 2001a, 2001b), not for less frequent mutations. Consequently, it is likely that the true hereditary fraction exceeds 4%. Further, BRCA2 mutations have demonstrated to have no significant occurrence in inherited epithelial ovarian cancer in Norway (Møller *et al*, 2001a).

In 1995, Ford *et al* (1995) stated that the frequency of BRCA1 mutation carriers in the general population was low. Based on data from population-based studies, they estimated the frequency of BRCA1 mutations in the British population (England and Wales) to be 0.06%. In 1997, Whittemore *et al* estimated the prevalence of BRCA1 mutations in the general US population to be 0.14% (Whittemore *et al*, 1997). In the Jewish population, the frequency of mutations in BRCA1 and BRCA2 at large is about 2% (Moslehi *et al*, 2000). In the present study, only two of the 1989 (0.10%) controls were mutation carriers.

Most ovarian carcinomas in women with BRCA1 mutations have been reported to be of serous histology (Risch *et al*, 2001). However, tumours of endometrioid and clear-cell histology are seen occasionally (Risch *et al*, 2001; Narod and Boyd, 2002). In the present study, 74% (14 of 19) of the tumours among the ovarian cancer mutation carriers were of serous histology. One tumour was of endometrioid histology, two were classified as mixed epithelial tumours and two were classified as undifferentiated carcinomas. Borderline tumours are rarely seen in women with BRCA mutations, although a few cases have been reported (Stratton *et al*, 1997; Borg *et al*, 1999). In the present study, no mutation carrier was discovered among the 190 patients with borderline tumours.

It is well documented that the mean age at diagnosis of ovarian cancer among mutation carriers is lower than among unselected patients, and that the prevalence of BRCA1 mutations decline with increasing age at diagnosis (Risch *et al*, 2001). In the present study, the median age at diagnosis among the mutation carriers was 49 years, 53 years among the noncarriers.

CA125, a high-molecular-weight glycoprotein, was first discovered in 1981, and has since demonstrated its value in monitoring the treatment of patients with ovarian cancer (Makar, 1993). CA125 has also proven its value in early detection of ovarian cancer. In 1988, Zurawski *et al* (1988) showed in a retrospective seroepidemiologic study from the Janus serum bank that half of the cases collected within the 18 months preceding diagnosis had elevated CA125 levels (more than 30 kU l^−1^). Owing to the relatively low sensitivity of this test, the combination of CA125 and ultrasound has been used for screening. However, intensive surveillance by the use of CA125 and ultrasound has not proven to be an effective means of diagnosing early-stage ovarian cancer in high-risk women (Liede *et al*, 2002). In the present study, CA125 showed to be a marker for ovarian cancer, but with a low sensitivity. CA125 was a fair or good test for ovarian cancer with a time perspective of about 1.5 years only. Two of the 19 ovarian cancer mutation carriers had elevated CA125.

In summary, this study showed that BRCA1 mutation carriers have a very high risk of ovarian cancer. However, since the prevalence of BRCA1 mutations in the Norwegian population was low, the proportion of ovarian cancers due to BRCA1 mutations seemed to be low. About 4% of the cases in the present study were mutation carriers. The sensitivity of using CA125 only as a screening test for ovarian cancer was low.
